# Weighted gene co-expression network analysis identifies molecular pathways and hub genes involved in broiler *White Striping* and *Wooden Breast* myopathies

**DOI:** 10.1038/s41598-021-81303-7

**Published:** 2021-01-19

**Authors:** Martina Bordini, Martina Zappaterra, Francesca Soglia, Massimiliano Petracci, Roberta Davoli

**Affiliations:** 1 Department of Agricultural and Food Sciences (DISTAL), Alma Mater Studiorum, University of Bologna, viale Fanin 46, 40127 Bologna, Italy; 2 Department of Agricultural and Food Sciences (DISTAL), Alma Mater Studiorum, University of Bologna, piazza Goidanich 60, 47521 Cesena, Italy

**Keywords:** Gene expression, Animal breeding

## Abstract

In recent years, the poultry industry has experienced an increased incidence of myopathies affecting breasts of fast-growing broilers, such as *White Striping* (WS) and *Wooden Breast* (WB) defects. To explore the molecular mechanisms and genes involved in WS and WB onset, we decided to perform a Weighted Gene Co-expression Network Analysis (WGCNA) using the gene expression profile and meat quality parameters of *Pectoralis major* muscles analysed in our previous study. Among the 212 modules identified by WGCNA, the red, darkred, midnightblue and paleturquoise4 modules were chosen for subsequent analysis. Functional analysis evidenced pathways involved in extracellular matrix (ECM) organization, collagen metabolism, cellular signaling and unfolded protein response. The hub gene analysis showed several genes coding for ECM components as the most interconnected nodes in the gene network (e.g. *COL4A1*, *COL4A2*, *LAMA2*, *LAMA4*, *FBLN5* and *FBN1*). In this regard, this study suggests that alterations in ECM composition could somehow activate the cascade of biological reactions that result in the growth-related myopathies onset, and the involvement of Collagen IV alterations in activating the endoplasmic reticulum (ER) stress response may be hypothesized. Therefore, our findings provide further and innovative knowledge concerning the molecular mechanisms related to the breast abnormalities occurrence in modern broilers.

## Introduction

Over the past 10 years, the poultry meat industry has faced a remarkable increase in the incidence of muscle disorders in commercial broilers, known as growth-related myopathies: White Striping (WS), Wooden Breast (WB) and Spaghetti Meat^1^. These defects are responsible for significant economic losses for the poultry industry, due to the impairment of the nutritional and technological quality of the affected fillets^2^. Therefore, several studies were carried out to identify the metabolic pathways especially involved in the onset of WS and WB, which have shown a complex etiology and a polygenic inheritance of these myopathies.

Since WS and WB share similar histological features, several Authors have hypothesized that these defects may share a common underlying mechanism^2–4^. In particular, breast muscles affected by WS and WB are characterized by degenerative lesions (resulting in atrophic fibers), occasional regenerative processes, variability in fibers cross-sectional area, fibrosis, lipidosis and infiltrations of inflammatory cells^1,4^. Several studies found that both these defects often coexist within the same fillet^4–6^. Besides, numerous studies have demonstrated that the incidence of emerging myopathies has a strong genetic determinism, as shown by the moderate (h^2^ = 0.34) to high (h^2^ = 0.65) hereditability values observed in high-yielding chickens^7,8^, thus suggesting that the prevalence of these growth-related abnormalities could be related to unfavorable correlated responses to broiler selection criteria adopted in modern broilers^2,9^.

Within this context, transcriptomic and gene expression studies can be helpful to improve knowledge concerning myodegenerative defects. In this regard, recent studies conducted in order to investigate the differentially expressed genes in abnormal *vs* normal fillets have evidenced pathway related to several functional categories, such as muscle development, glucose and polysaccharides metabolism, intracellular transport, immune response, inflammation, response to reactive oxygen species and blood vessels morphogenesis^5,10–12^. These functional categories are often involved in case of compromised blood and oxygen supply, suggesting that tissues affected by modern myopathies could be characterized by hypoxic conditions^5,10,13^. Within this context, it has been speculated that hypoxia could be one of the possible major causative processes underlying the occurrence of these muscular disorders^2^. According to this scenario, Hoving-Bolink et al.^14^ and Soglia et al.^1^ reported that the hypertrophy induced by the genetic selection carried out in fast-growing broiler led to a reduced capillary density, which may result in an impaired oxygen and nutrient supply to the muscle. However, it is not certain whether this chain of events could be the primary cause or rather contributes to worsen the phenotypes found in affected animals. Thus, the underlying causes of these breast abnormalities remain currently unclear.

The present study was aimed to investigate the molecular and biological processes involved in the onset of the phenotypes characterizing these conditions. In detail, this work was performed to better understand the molecular pathways that underly these modern abnormalities by evaluating the association between groups of co-expressed genes and the most important phenotypes associated to WS and WB defects. For this purpose, the gene expression profile and the technological and quality traits measured on affected and unaffected broilers in our previous study^5^ were analyzed. In particular, the data obtained by Zambonelli et al.^5^ were used to construct a gene co-expression network that may identify clusters of candidate genes likely involved in WS and WB occurrence, with the aim of improving the knowledge regarding the molecular cascade of events associated with the development of the modern growth-related myopathies.

## Results

### Weighted gene co-expression network analysis (WGCNA)

**Figure 1:**
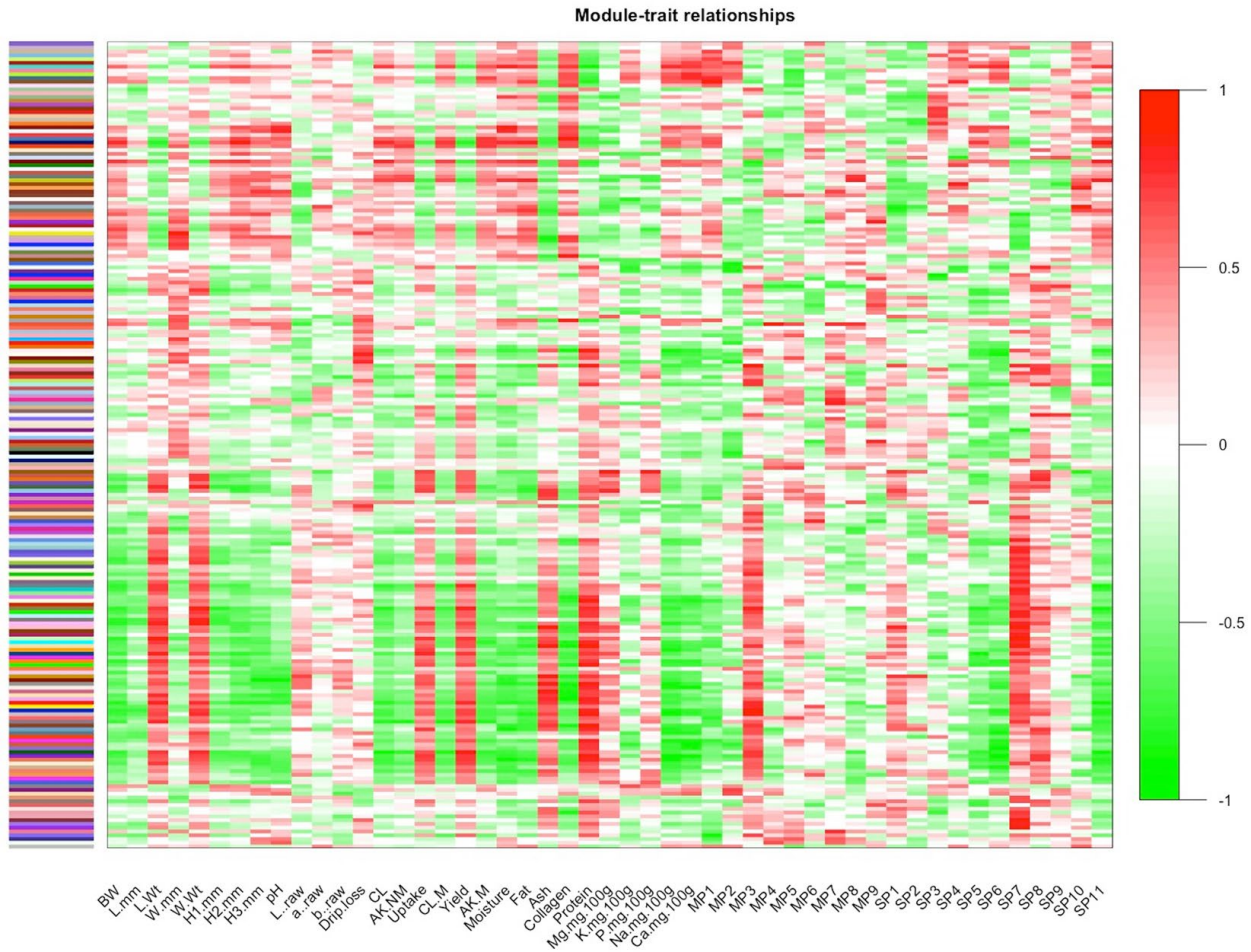
Heatmap representing associations between modules and traits (Module-trait relationships). Traits are reported on the x-axis, and modules are reported on the y-axis. The heatmap is color-coded by correlation values: green color represents a negative correlation, while red color represents a positive correlation. In the figure, “M” stands for Marinated meat, “NM” for Not Marinated, “MP” for Myofibrillar Protein, and “SP” for Sarcoplasmic protein. The complete explanation of the traits on the x-axis is reported in Zambonelli et al.^5^.

The weighted gene co-expression network was constructed using the microarray data of the *Pectoralis major* muscle expression profiling and the meat quality parameters reported in our previous study^5^. As a result of network construction, the WGCNA analysis found 212 modules (i.e. cluster of co-expressed genes), identified by a different color, with sizes ranging from 1889 to 30 probes. The 212 gene modules identified by WGCNA are shown by cluster dendrogram (Supplementary Figure S1), in which the branches correspond to modules and each leaf in the branch represents one probe. Among the 18,308 probes analysed, only 24 were assigned to any modules. Furthermore, the WGCNA R package allowed us to quantify correlation values between the genes of each module and the considered phenotypes, assessing in this way the module-trait association. In this regard, Fig. 1 shows the associations between modules and traits using a heatmap plot, which graphically represents Pearson’s correlation coefficients measured between each single module and trait (Supplementary Table S1), as described in “Methods” section.

Considering the huge amount of data obtained, we decided to focus on a few phenotypes and modules for subsequent analyses. In particular, we mainly considered those modules that were more significantly correlated with the 9 traits that according to Zambonelli et al.^5^ were most significantly associated with WS and WB defects (*P* < 0.0001 in the Student’s *t-test*), i.e. breast weight (BW), the height of the breast measured at a half distance of the breast length (H2.mm), cooking loss (CL), breast length (L.mm), breast width (W.mm), marinade uptake (Uptake), processing yield (Yield), protein content (Protein) and Allo-Kramer shear force (AK.M). Among them, the analysis of the module-trait associations allowed us to highlight that BW, H2.mm and CL showed a similar trend of correlation with modules (as shown by the similar alternation of colors for these three traits in the heatmap plot) (Fig. 1), which means that each one of these three traits shared the same most significantly correlated modules. Assuming this result suitable and appropriate for the purpose of our research, we decided that the approach to use in this study should be focusing on the traits showing the same trend of significant correlations with modules (i.e. BW, H2.mm and CL), especially considering that these three traits are those most associated with modern myopathies, as shown by Zambonelli et al.^5^. Therefore, we focused our analysis mainly on modules more significantly related to BW, H2.mm, and CL.

Moreover, on the basis of the high associations of the traits BW, H2.mm and CL with WS and WB myopathies, and considering their similar trend of correlations with the gene modules (pointed out by the same most significantly modules shared by these three traits), we decided to consider all together these traits as if they were a comprehensive phenotype: a complex “macro-trait”. Next, we evaluated the relationship between each module and the phenotypes included in the “macro-trait”, following the criteria reported in “Methods” section. We found that four modules (red, darkred, paleturquoise4 and midnightblue), which respectively include 256, 177, 43 and 143 genes, exhibited similar characteristics of correlation and significance in BW, H2.mm and CL (absolute correlation value r > 0.7 and *P*-value < 0.01; Table 1). Notably, among them, two modules (red and paleturquoise4) were negatively correlated with the phenotypes in the “macro-traits” (r < − 0.72, *P*-value < 0.007; Table 1), and two modules (darkred and midnightblue) positively correlated with the “macro-trait” (r > 0.74, *P*-value < 0.005; Table 1). Futhermore, we measured the module significance (MS), which represent the average absolute value of correlation (i.e. gene significance) for all genes in a given module. The module significance values of selected modules are shown in Table 1, while all module significance values detected by WGCNA are reported in Supplementary Table S5.

**Table 1:** Pearson’s correlation values (r) between the modules most significantly associated with the macro-trait and phenotypes belonging to the macro-trait: breast weight (BW), middle height (H2.mm), and cooking loss (CL).

Modules	BW		H2.mm		CL	
	r	MS	r	MS	r	MS
Darkred	0.76*	0.65	0.74*	0.63	0.79*	0.68
Midnightblue	0.79*	0.66	0.85**	0.71	0.77**	0.64
Red	− 0.83**	0.69	− 0.82*	0.68	− 0.83**	0.68
Paleturquoise4	− 0.80*	0.67	− 0.72*	0.61	− 0.76*	0.64

Also, significance values (MS) of each selected module are reported.

*P < 0.01; **P < 0.001.

### Functional enrichment analysis of interesting modules

The four modules that met the criteria previously reported and discussed in “Methods” section (i.e. red, darkred, paleturquoise4 and midnightblue) were functionally annotated with DAVID tools. This analysis was performed considering the red, darkred, paleturquoise4 and midnightblue not only as individual modules but also combining them into one unified and comprehensive module (which will be referred from now on as "macro-module"), more specifically by analysing in DAVID both the gene lists of each individual module and one single list obtained by merging the gene lists of individual modules. The choice to analyse the four modules all together was aimed at obtaining additional biological information about the interaction between the networks of genes most related to the phenotypes of interest (the macro-trait), and possible functional relationships existing between these groups of co-expressed genes identified by WGCNA. The detailed results obtained from the DAVID functional classification of the macro-module and each selected module are reported in Supplementary Table S2. Considering the macro-module, Table 2 reports functional categories identified as significant by DAVID, such as extracellular matrix organization, collagen catabolic process, unfolded protein binding and platelet-derived growth factor binding.

**Table 2:** DAVID functional clustering obtained considering the macro-module containing darkred, midnightblue, paleturquoise4 and red modules.

Category	Term	Gene count	Benjamini adjusted *P*-value
GOTERM_BP	GO:0030198 ~ extracellular matrix organization	48	8.18E-06
	GO:0030574 ~ collagen catabolic process	18	0.040
GOTERM_MF	GO:0051082 ~ unfolded protein binding	26	0.010
	GO:0048407 ~ platelet-derived growth factor binding	8	0.012
KEGG	hsa05215:Prostate cancer	24	0.002
	hsa04512:ECM-receptor interaction	20	0.046
	hsa04916:melanogenesis	27	0.001
	hsa03010:ribosome	28	0.039
	hsa04015:Rap1 signaling pathway	38	0.044
UP_KEYWODS	Chaperone	32	0.013
	Ehlers-Danlos syndrome	9	2.00E−04
	Collagen	22	0.001
	Hydroxylation	18	0.031
	Glycoprotein	452	0.001
	Membrane	734	1.64E−05
	Nucleotide-binding	196	0.003
	EGF-like domain	34	0.040
	Activator	89	5.14E−04

### Functional analysis and detection of hub genes

**Figure 2:**
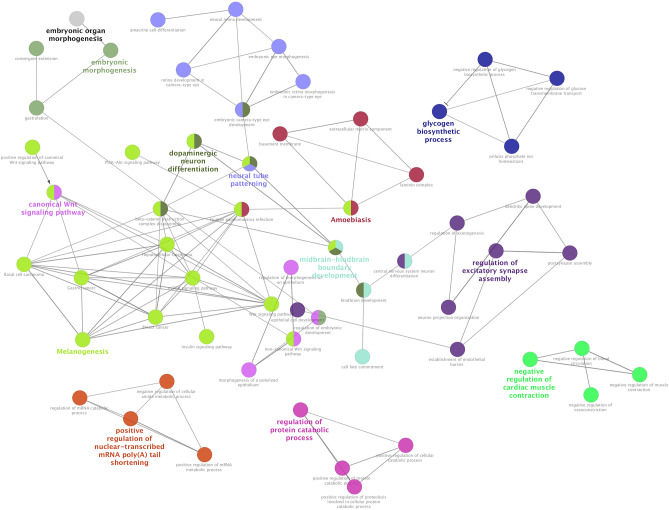
Macro-module functional network. This image shows the most significant functional categories identified by ClueGO Cytoscape plugin and how terms are grouped and interact with each other. Different groups of functional terms are distinguished from different colors. Terms that belong to two different groups have both colors of annotation groups to which they belong. The name in bold indicates the leading group term, according to the highest significance related to Benjamini-Hochberg.

Considering the macro-module, Fig. 2 shows functional terms and their interconnections (how they are interconnected) identified by ClueGO. In particular, these results have shown several functional categories related to cellular components and biological processes, such as basement membrane, laminin complex, establishment of endothelial barrier, positive regulation of protein catabolic process, Wnt and PI3k/Akt/mTOR signalling pathways (Table 3). The detailed results of ClueGO analysis are reported in Supplementary Table S3. Furthermore, Table 3 shows which terms are significant for both the two methods used for the functional annotation: DAVID tool and ClueGO Cytoscape plugin. Interestingly, the extracellular matrix components were found significant for both the two methods, along with the metabolic pathways related to protein catabolism.

**Table 3:** Comparison between ClueGO and DAVID results.

Clusters of functional categories	ClueGO	DAVID
Embryonic morphogenesis (gastrulation, regulation of embryonic development)	✓	
Regulation of muscle contraction (negative regulation of muscle contraction, vasoconstriction and blood circulation)	✓	
Nervous system development (midbrain-hindbrain boundary development, cell fate commitment, regulation of axonogenesis, neural tube patterning, neural retina development, dopaminergic neuron differentiation)	✓	
Glycogen biosynthetic process (negative regulation of glucose transmembrane transport, negative regulation of biosynthetic process)	✓	
Extracellular matrix component (basement membrane, laminin complex)	✓	✓
Catabolic processes (positive regulation of cellular catabolic process, positive regulation of protein catabolic process)	✓	✓
Multisystem development (epithelial cell development, establishment of endothelial barrier, dendritic spine development)	✓	✓
Regulation of gene transcription (regulation of mRNA catabolic process, positive regulation of mRNA metabolic process, positive regulation of nuclear-transcribed mRNA poly(A) tail shortening)	✓	✓
Signaling pathways (mTOR signaling pathway, PI3K-Akt signaling pathway, Wnt signaling pathway, insulin signaling pathway)	✓	✓
Melanogenesis	✓	✓
Diseases (amoebiasis, basal cell carcinoma, breast cancer, gastric cancer)	✓	✓

In this table, the clusters of functional terms identified by the ClueGO analysis are reported. The ticks show the clusters of functional categories identified as significant clusters with both methods (ClueGO and DAVID). Benjamini-adjusted *P*-value < 0.05 was chosen as the significance threshold.

The cytoHubba Cytoscape plugin was used to better understand how all genes belonging to individual selected modules, that on the whole represent the macro-module, interact with each others. Moreover, cytoHubba was used to identify the most interconnected genes in the considered network, thus suggesting their biological involvement in the onset of traits significantly related to modern abnormalities. In this regard, cytoHubba allowed us to detect hub genes (i.e. genes that had the most connections to other genes of the network) and their subnetwork. Considering the macro-module, cytoHubba plugin detected 10 genes that can be considered as hubs: *Collagen type IV alpha 1 chain* (*COL4A1*)*, Laminin subunit alpha 4* (*LAMA4*), *Collagen type IV alpha 2 chain* (*COL4A2*), *Fibulin 5* (*FBLN5*), *Fibrillin 1* (*FBN1*), *Collagen type XV alpha 15 chain* (*COL15A1*), *Leucine rich repeat containing 32* (*LRRC32*), *Plexin domain containing 2* (*PLXDC2*), *Laminin subunit alpha 2* (*LAMA2*) and *Laminin subunit gamma 1* (*LAMC1*). The correlation values (i.e. Gene Significance) between genes identified as hubs by cytoHubba and the traits considered for the present study are reported in Supplementary Table S6.

**Figure 3:**
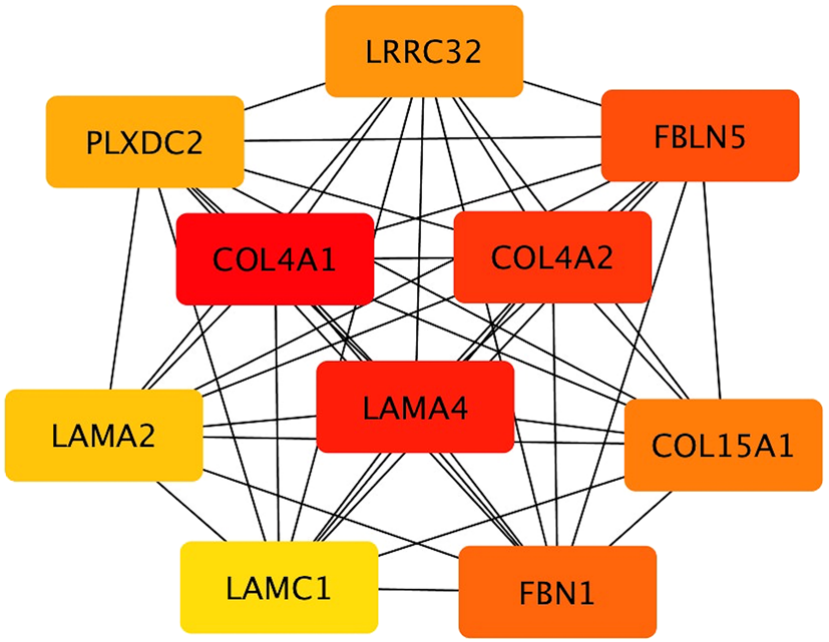
Top 10 hub genes in the macro-module identified by CytoHubba Cytoscape plugin. Genes with the highest number of connections in the network are detected as hub genes. The image shows the degree of importance of hubs through a color scale ranging from red (the most important) to yellow (less important).

**Figure 4:**
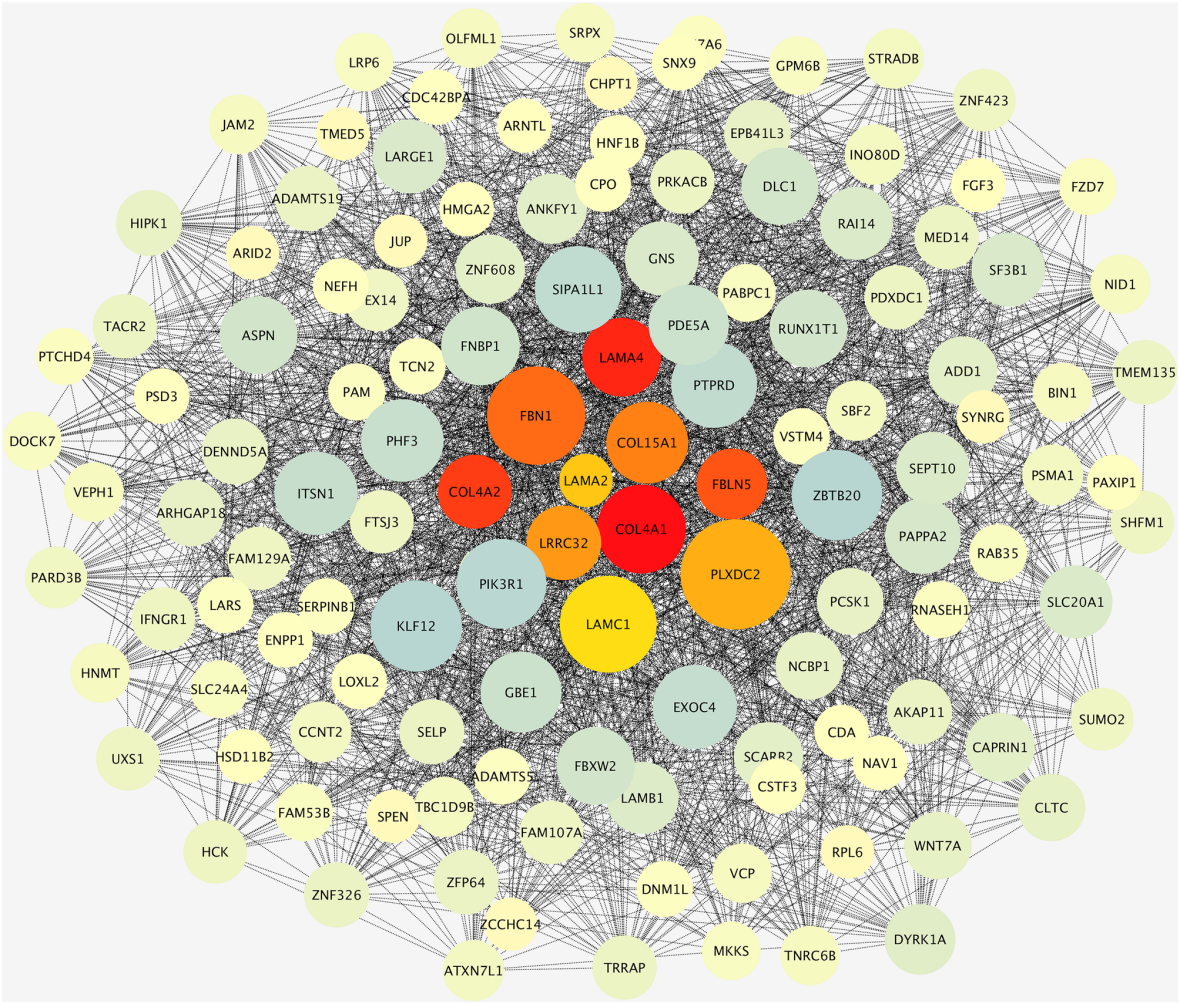
Subnetwork of hub genes extracted from CytoHubba. The figure shows the interconnections between hub genes and other genes in the macro-module.

Intriguingly, most of the genes identified as hubs are components of the extracellular matrix, with particular reference to collagen proteins and laminins. The output of cytoHubba analysis is reported in Fig. 3. Besides, Fig. 4 shows the subnetwork of hub genes, which represents how they are interconnected with other genes of the macro-module.

## Discussion

WS and WB abnormalities are responsible for a substantial impairment of both the technological properties and the nutritional value of fast-growing broiler fillets^2^. Considering their detrimental effect on the poultry industry, an improvement in the knowledge regarding the molecular pathways involved in the onset of these modern myopathies can be extremely important. Within this scenario, our study is intended to investigate which molecular pathways may be involved in the occurrence of the phenotypes characterizing the WS and WB abnormalities.

To investigate more in depth the biological processes involved in these conditions, we considered useful to perform a WGCNA analysis, in order to assess the differences in the gene expression profiles in relation to the phenotypic variability of quality and morphological traits measured in normal and abnormal fillets. Particularly, a weighted gene co-expression analysis was used to evaluate the association between networks of co-expressed genes and the most important phenotypes related to WS and WB defects, referring to the traits more associated with the abnormal condition. The results of these associations enabled us to identify genes that can be involved in the metabolic pathways of *Pectoralis major* muscle, and that may be associated with phenotypes occurring in the presence of growth-related myopathies. In this regard, we assumed worthwhile to consider a single comprehensive macro-trait containing the traits BW, H2.mm, and CL that were the most significantly associated with the breast abnormalities and that shared the same most significantly related gene modules, as described in “Results” and “Methods” sections. Therefore, the study allowed us to understand how the phenotypes of “macro-trait” are linked to the network of co-expressed genes.

Among the modules most significantly related to the macro-trait, the red, darkred, midnightblue and paleturquoise4 modules were considered for the functional characterization. By using DAVID tool we found that genes belonging to these modules are significantly associated with GO terms related to the extracellular matrix (ECM) organization. These findings agree with the results of previous studies that evidenced an upregulation of several genes directly involved in the ECM remodeling^10,15,16^. Among them, Papah et al.^15^ and Pampouille et al.^16^ have found that *Platelet-derived growth factor receptor alpha* (*PDGFRA*) was upregulated in broiler fillets affected by WB defect. Additionally, the same gene was one of the candidate genes potentially involved in the onset of these defects as detected by Pampouille et al.^11^. According to these findings, we found that genes of macro-module were enriched with molecular function related to platelet-derived growth factor binding. This result agrees with the data reported by Papah et al.^15^, who described that an increased expression of genes belonging to molecular pathways related to platelet-derived growth factor results in aberrant deposition of the extracellular matrix components. This state in turn leads to a fibrotic condition, which is one of the main distinctive characteristics of WB fillets^17,18^. In addition, Velleman^19^ reports that the ECM organization is deeply involved in the manifestation of fibrosis affecting abnormal breasts. In this regard, the profound alteration and the fibrosis observed in previous researches were in agreement with our results that suggest abnormalities in the ECM organization. Among the functional terms identified in our work, there is also the ECM-receptor interaction. The presence of this complex pathway, other than highlighting a key role of the ECM in the altered muscle condition, suggests that some of the pathological changes characterizing muscle abnormalities could be coordinated by the ECM, as already assumed by Pampouille et al.^11^.

Concerning the upregulation of genes involved in the ECM remodeling pathway, in literature it is reported that the activation of Matrix Metalloproteinase (MMPs) genes is linked to modifications in the ECM composition^20^. Mutryn et al.^10^ have identified Matrix Metalloproteinases-2 as an upregulated gene in birds affected by WB. The type 2 of MMPs is typically associated with collagen degradation, with particular reference to collagen type IV^10^. It is worth noting that these insights agree with another functional class identified in our study: the “collagen catabolic process”. Additionally, ClueGO, has also identified the functional category called “positive regulation of protein catabolic process”, and this result is in accordance with the relevant decrease in protein content that characterized abnormal fillets, as widely described in the literature^2^.

Results from DAVID also evidenced pathways probably related to cellular stress response, as suggested by the presence of the functional categories “unfolded protein binding” and “Chaperone”. This result may indicate the activation of the unfolded protein response (UPR), which represents a cellular stress response following the accumulation of unfolded or misfolded proteins in the endoplasmic reticulum (ER), called sarcoplasmic reticulum in skeletal muscle cells. The UPR pathways are often activated in the presence of disease associated with ECM alterations^21^. Notably, Lake et al.^22^ have evidenced that 2–3 weeks old birds shown differentially expressed genes linked to the ER stress and the UPR activation, suggesting their involvement in the early onset of growth-related myopathies in broilers.

Moreover, functional enrichment analysis of the macro-modules genes showed many GO terms functionally associated with signaling pathways, such as Wnt and PI3k/Akt/mTOR signaling pathways. In literature, it has been speculated that the activation of the Wnt signaling pathway can leads to fibrosis condition, by exhausting the satellite cell activity^23,24^. In this scenario, Phillips et al.^24^ have supposed that excessive stimulation of the Wnt signal, through mTOR pathways, could potentially result in WB.

The CytoHubba analysis allowed us to identify genes playing important roles in the network analyzed in our study (the macro-module). In fact, since the nodes having many connections (i.e. hubs) could play a regulatory role in biological networks, genes belonging to macro-module that are identified as hubs can be considered functionally important. Thus, considering the significant association between the macro-trait phenotypes and the presence of breast abnormalities, the genes belonging to the macro-module identified as hubs in the present study may be involved in the molecular processes underlying these conditions. Notably, the results obtained in our study have shown that most of the genes identified as hubs encode for ECM components, such as *LAMA2*, *LAMA4*, *FBN1, FBLN5,* and *COL4A1/COL4A2*. In this respect, here the importance of ECM function and composition, as suggested by results obtained from functional characterization and hub genes analysis has been evidenced.

In support of this finding, the relevant role of ECM in maintaining the structure and integrity of skeletal muscle is widely described in the literature. Indeed, ECM is essential for tissue architecture and for regulating intercellular communications^25^. Alterations in key components of the ECM can lead to muscular abnormalities, as proven for muscular dystrophies in humans^26^.

Among the genes identified as hubs, *LAMA2* and *LAMA4* encode for a group of ECM fundamental components: the laminins^27^. Specifically, laminins are the main non-collagenous glycoprotein constituting the basement membrane (BM)^28^. This type of glycoproteins is known to be involved in myoblast proliferation and differentiation, and to be associated with myopathies in humans^25^. In particular, mutations in *LAMA2* result in a type of muscular congenital dystrophy: the “Laminin-α2 chain-deficient congenital muscular dystrophy” or “LAMA2-CMD”^29,30^. Besides, studies carried out in *LAMA2* deficiency-induced mice (*LAMA2*^−/−^) have shown that the complete deficiency of laminin-α2 chain determines a severe form of LAMA2-CMD condition^31^.

Regarding the laminin-alpha4, its exact function is not well known yet. However, *LAMA4* seems to promote migration, proliferation, and survival of endothelial cells in humans^27^.

*FBN1* is a gene that codes for fibrillin-1: another central component of ECM. In humans, fibrillin-1 contributes to the maintenance of the functionality and stability of connective tissue^32^. Given that, mutations in the human *FBN1* gene are associated with connective tissue disorders. The most common disease linked to FBN1 mutations is the Marfan syndrome, an autosomal dominant multi-systemic disorder of connective tissue^32,33^. Similarly, the protein encoded by *FBLN5* belongs to a family of proteins called “fibulins”, which are involved in several functions of the ECM. More in detail, fibulin-5 seems to play a relevant role in the elastic fiber assembly and to provide flexibility to the connective tissues^34^.

Among the hub genes detected, we have found *COL4A1* and *COL4A2* as the most interconnected hub nodes in the macro-module network. These genes are characterized by an high degree of conservation across species, which suggests their biological importance for normal function of organisms^35^. They encode for protein forming the Collagen type IV heterotrimers ([a1(IV)]2a2(IV)): a non-fibrillar collagen which represents the major component of the BM of many tissues (e.g. skeletal muscles, epithelial and endothelial cells)^36–38^. Several Authors have evidenced that some mutations in human genes coding for COL4A1 and COL4A2 proteins cause multi-system disorders including muscular abnormalities, known as “COL4A1/COL4A2 syndrome”^35,36,38^. In the view of the involvement of these genes in myopatic conditions, they were deeply studied in human and animal models (e.g. mice) to investigate pathogenic mechanisms underlying Collagen type IV disorders. In contrast, to date, little is known about *COL4A1* and *COL4A2* genes in chickens. Nevertheless, based on their possible involvement in breast abnormalities onset, other types of collagen are being recently studied in fast-growing broilers: Collagen type VI^16^ and III^39^.

In this context, it is worth mentioning the myopathic changes that characterized human and mice skeletal muscles affected by COL4A1/COL4A2 syndrome. Histological examination of the skeletal muscle sample from individuals who had *COL4A1* mutations revealed features very similar to the histopathological changes observed in broiler breasts affected by WS and WB defects including patchy myopathic lesions consisting in myofiber size variation, endomysial fibrosis, focal fatty replacement, non-peripheral nuclei, cluster of necrotic and regenerating myofibers and infiltration of inflammatory cells^38,40^. Similarly to what is observed in WS and WB fillets, morphologic characteristics of skeletal muscle affected by COL4A1 disorders appear to be more pronounced with age, suggesting that myopathy caused by mutations in the *COL4A1* gene in human is progressive^38^. This condition represents an additional similarity to the growth-related myopathies affecting chickens, since a progression with age/growth is assumed for these breast muscle abnormalities^4,41^. Moreover, COL4A1 mutant human and mice muscles present lesions at different stages of the same disease processes, as identified for the broiler breast myopathies^42^.

In humans, the pathogenic mechanisms of COL4A1-related myopathy are linked to a reduction of the extracellular secretion of the mutant Collagen type IV, as shown by Kuo et al.^35^. In particular, several *COL4A1* polymorphisms, both in humans and animal models, were associated with the occurrence of an ER stress condition and the activation of the UPR^40^. These findings agree with our results. Indeed, DAVID analysis has found the “unfolded protein binding” and the “Chaperon” among significant functional terms, thus suggesting possible UPR activations. Interestingly, Lake et al.^22^ have evidenced that ER stress occurs in the early stage of WB abnormality, and they have hypothesized its involvement in the onset of this myopathic condition. Therefore, we hypothesize that the intracytoplasmic accumulation of mutant Collagen type IV could be implicated in the onset of ER stress condition of muscular cells, resulting in an alteration of the ECM structure and apoptosis process in abnormal breast muscles.

The ER stress would also explain the dysregulation in calcium homeostasis observed in abnormal breasts, already in 2- and 3-weeks old broilers^20^. In fact, the ER represents a dynamic Ca^2+^ store, and failure in ER homeostasis leads to several conditions well described in abnormal breasts, such as dysregulation of calcium homeostasis, UPR activation, and cellular apoptosis^43,44^. According to this, our previous results evidenced the up-regulation of genes linked to calcium homeostasis, with particular reference to those involved in processes leading to increased intracellular Ca^2+^ (i.e. purinergic receptor pathways)^5^. Thus, the failure in the ER activities (e.g. the preservation of normal Ca^2+^ storage and release) could explain the alteration in calcium signaling pathways found by Zambonelli et al.^5^. Furthermore, ER dysfunction has important consequences for insulin secretion and glucose metabolism alteration^44,45^, which agrees with the modified glycolytic enzymes expression previously found in abnormal breasts^5^. These statements could also explain the severely altered glucose metabolism identified by Lake and Abasht^20^ and Baldi et al.^46^ in broilers affected by WB. In addition, the same Authors pointed out the importance of the endothelial cell damage and the apoptosis promoted by UPR in the occurrence of the growth-related muscular abnormalities in broilers, in accordance with the potential pathogenic mechanisms of COL4A1-related myopathies. Indeed, human and mice COL4A1-mutant muscles have been associated with the endothelial cell damage in muscular capillaries. More in detail, it was demonstrated the contribution of vascular defects to the development of COL4A1-related myopathies, probably due to failure of the type IV collagen secretion from endothelial cells and its intracellular accumulation, resulting in the ER stress condition^21,35,38,40^. Interestingly, vascular damage represents the first lesion appearing before the development of myopathic alterations in broilers’ abnormal breasts^47^. Also, Papah et al.^15^ considered these conditions critical for the initiation of pathology, according to the vascular defects discovered in the COL4A1 mutant muscles^40^. Therefore, these assumptions agree with our results and strengthen the hypothesis that endothelial damage could be one of the leading mechanisms in growth-related abnormalities onset.

Based on these considerations and bearing in mind the relevant role of hub genes in biological networks, our findings suggest the possible involvement of certain ECM components in the occurrence of broilers growth-related muscular abnormalities. Moreover, considering the strong similarities between the histological and molecular features that characterize COL4A1-related disorders in humans and growth-related myopathies in broilers, a probable involvement of Collagen type IV in the onset of WS and WB could be hypothesized. In particular, we assume that the ECM impairment, along with the endothelial alteration could contribute or be one of the primary causes of WS and WB occurrence.

In conclusion, we hypothesize that ECM disorders could have an important role in the pathogenesis of WS and WB. In particular, we suggest that ECM disorders may explain the alterations in muscular metabolism of abnormal breasts, with special reference to glucose metabolism. Among the possible causes leading to ECM disorders, the involvement of Collagen type IV alterations in activating the ER stress response could be supposed. Therefore, it might be speculated that ECM disorders could somehow trigger or be involved in the cascade of the biological reactions that lead to the occurrence of those phenotypes that characterize the growth-related muscular abnormalities. Although additional studies are needed, the present research provides further elements of knowledge in the comprehension of the molecular mechanisms related to the occurrence of breast abnormalities in modern broilers. Future research works could be carried out to investigate the possible effects of *COL4A1* mutations in chickens and more generally the role of ECM disorders in the etiology and pathogenesis of WS and WB myopathies.

## Methods

### Data collection

The present study was carried out using the data obtained from our previous microarray analysis^5^ in which were tested and compared the gene expression profiles of 12 samples of broiler *Pectoralis major* muscles (6 having macroscopically normal appearance *vs* 6 severely affected by WS/WB defect). Concerning the traits considered for our analysis, all the phenotypes analyzed by Zambonelli et al.^5^ were employed for the WGCNA construction (Supplementary Table S4). In particular, the meat quality traits considered in our previous research, and then used for the present study, include both morphological and technological traits, along with measurements related to the chemical composition of chicken meat. The detailed sample selection and preparation is reported in Zambonelli et al.^5^.

For each sample, the expression levels of 18,308 probes were used to construct the co-expression network and identify groups of genes related to the 49 external traits considered for this work. In particular, the WGCNA analysis enabled us to identify groups of genes characterized by a similar expression profile (i.e. modules of co-expressed genes) and to investigate their relationship with the whole set of phenotypes described in Zambonelli et al.^5^.

### Co-expression network analysis

For the present study, a Weighted Gene Co-expression Network Analysis was performed using the WGCNA R package^48^ in order to assess the relationships between clusters of co-expressed genes and phenotypes related to modern myopathies. At first, the network was constructed grouping the functionally correlated genes into modules based on the pairwise correlations, corresponding to their similar gene expression level^48^.

To construct the network of co-expressed genes and to cluster genes that exhibit similar expression patterns, the WGCNA package built an adjacency matrix in which the nodes of the network correspond to gene expression profiles, and edges between genes are determined by the pairwise correlations between gene expressions, calculated using the Pearson’s correlation^48^. First, it was necessary to find an optimal soft-thresholding power to transform the co-expression similarity into adjacency. Thus, after performing the analysis of network topology for several soft-thresholding parameters, power 10 was chosen as the soft-thresholding power to reach a scale-free topology index (Supplementary Figure S2). Then, the gene network was constructed using the blockwiseModules function, and modules of co-expressed genes were detected by using hierarchical clustering^48^ (Supplementary Figure S1). In the blockwiseModules function we have chosen 2 for DeepSplit parameter and 30 for the minimum module size^48^.

Once identified groups of genes characterized by a similar trend of expression profile (i.e. modules), it was necessary to detect modules that were most significantly related to the measured traits of interest. In this regard, the association values between modules and traits were quantified using Pearson’s correlation. In particular, to identify the module-trait relationship, the WGCNA package determined the expression value of each module (i.e. module eigengene; ME) using the principal component analysis. Indeed, the ME can be considered representative for the gene expression profile of the corresponding module^48^. This approach allowed us to calculate Pearson’s correlations between each module eigengene and trait, and thus to identify the module-trait relationship.

### Identification of significant traits and modules

Among the 49 traits considered for the present study, we have decided to focus on traits that in our previous work were found to be the most associated with the occurrence of WS/WB muscle abnormalities, with a *P-value* less than 0.0001 in the Student’s *t-test*^5^. Among them, we considered breast weight (BW), middle height (H2.mm), and cooking loss (CL) as interesting for further investigations since they have shown a similar trend of correlation values with modules identified by the WGCNA analysis. Besides, considering the similar trend of correlations and the high significance values that characterized these phenotypes, the BW, H2.mm and CL traits were considered as a complex “macro-trait”, as well described in “Results” section.

Subsequently, among the groups of co-expressed genes detected in this study, we wanted to point out the most significant modules with the aim to perform further analysis and investigate their biological networks. In particular, we have chosen to focus on modules that (i) were shared as modules significantly related by the phenotypes belonging to the macro-trait (i.e. BW, H2.mm, and CL); (ii) were the most significantly correlated with the macro-trait (*P* < 0.01); (iii) were characterized by an absolute value of module-trait correlation higher than 0.7 (in agreement with Pampouille et al.^16^). Thus, the most significant modules were identified using the requirements previously described. Furthermore, we decided to analyze the most significant modules not only individually, but also as a single unified module (named as “macro-module”), in order to investigate the interactions between genes belonging to the modules most significantly associated with the macro-trait.

### DAVID tools functional enrichment analysis

Functional enrichment analysis was carried out by the Database for Annotation, Visualization and Integrated Discovery (DAVID) version 6.8^49^, using the DAVID default background. In particular, the Functional Annotation Clustering function was used with the aim to point out the most relevant functional terms associated with a given gene list. GO terms, KEGG pathways^50^ and UniProt Keywords included in the DAVID Knowledgebase were considered for the functional characterization. Among the GO terms categories, the biological processes and molecular functions have been considered for the DAVID enrichment analysis. This analysis was performed on both gene lists of individual modules and the gene list of the macro-module, as deeply described in the Results section. In this analysis, a Benjamini-adjusted *P-value* of 0.05 was chosen as the significance threshold to identify the most significant functional categories.

### ClueGO functional characterization

Another functional analysis was conducted using ClueGO^51^, a Cytoscape plugin (Cytoscape software 3.7.2)^52^. In particular, we decided to deeper analyze the functional characterization of genes belonging to the macro-module and to explore how functional terms are interconnected between each other^51^. In this regard, the ClueGO plugin allowed us to visualize the biological terms that characterized this cluster of genes highly associated with the macro-trait, which was in turn significantly related to modern myopathies. In detail, this plugin creates an “annotation network” in which similar functional terms are grouped by the same color, and it shows how these terms are interconnected to each other. In the ClueGO analysis, the *P-value* was adjusted using the Benjamini–Hochberg method and a threshold for significance of *P* < 0.05 was chosen.

### Identification of hub genes

**Figure 5:**
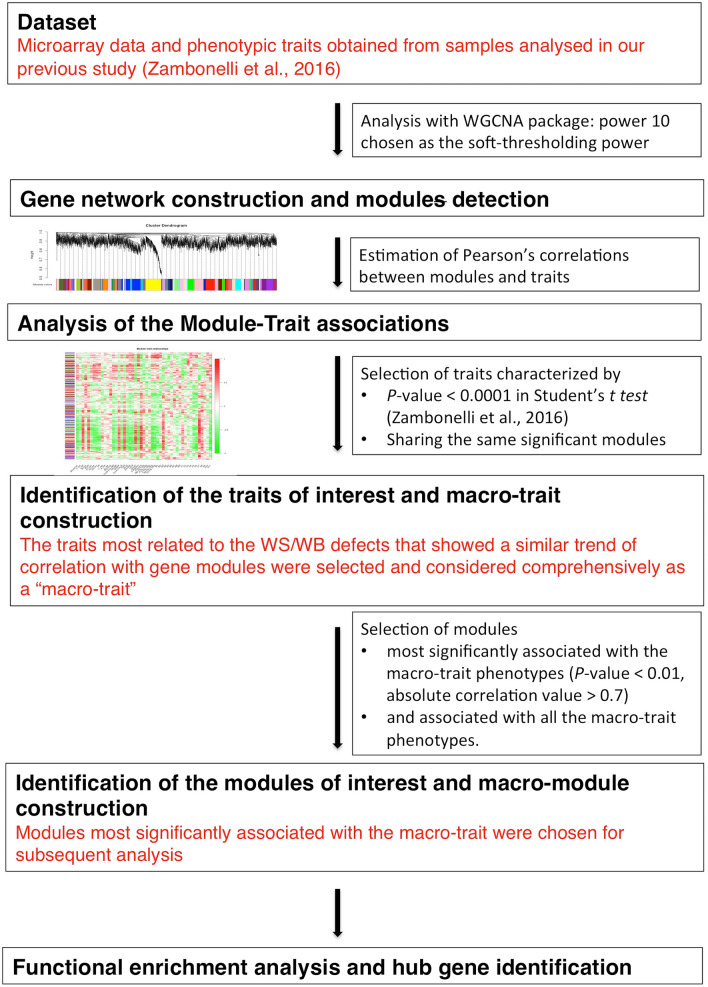
Flowchart of the performed analysis. The scheme shows the steps of the gene network construction, identification of traits and modules of interest and analysis of the data obtained by WGCNA.

CytoHubba Cytoscape plugin was used to explore the hub genes network^53^. This plugin provides tools for exploring important nodes in biological networks by employing several algorithms to identify hub nodes and how they are interconnected with other genes. In our study, to detect the hubs we have used the MCC algorithm, which calculates the connectivity of each gene in the network and identifies those that are the most interconnected, i.e. hub genes^52^. Then, the software has created a network in which hub nodes are colored in accordance with their importance (calculated by the MCC algorithm): from red for the most important, to yellow for less important. Besides, this plugin has provided the hub genes subnetwork. In particular, the subnetwork of essential nodes extracted by cytoHubba allowed us to visualize genes that directly interact with these top-ranked nodes, using the option “*check first stage node*”. To summarize the flowchart of this work, Fig. 5 shows all steps performed in our analysis.

## Supplementary Information

Supplementary Information.

Supplementary Table S1.

Supplementary Table S2.

Supplementary Table S3.

Supplementary Table S4.

Supplementary Table S5.

Supplementary Table S6.

## Data Availability

The microarray data analysed during the current study are available in the U.S. National Center for Biotechnology Information GEO database with the accession number GSE79276.

## References

[1] Soglia F, Mazzoni M, Petracci M (2019). Spotlight on avian pathology: Current growth-related breast meat abnormalities in broilers. Avian Pathol..

[2] Petracci M (2019). Wooden breast, white striping, and spaghetti meat: Causes, consequences and consumer perception of emerging broiler meat abnormalities. Compr. Rev. Food Sci. Food Saf..

[3] Kuttappan VA, Hargis BM, Owens CM (2016). White striping and woody breast myopathies in the modern poultry industry: A review. Poult. Sci..

[4] Griffin JR, Moraes L, Wick M, Lilburn MS (2018). Onset of white striping and progression into wooden breast as defined by myopathic changes underlying pectoralis major growth. Estimation of growth parameters as predictors for stage of myopathy progression. Avian Pathol..

[5] Zambonelli P (2016). Detection of differentially expressed genes in broiler pectoralis major muscle affected by White Striping–Wooden Breast myopathies. Poult. Sci..

[6] Bowker B, Zhuang H, Yoon SC, Tasoniero G, Lawrence K (2019). Relationships between attributes of Woody Breast and White Striping myopathies in commercially processed broiler breast meat. J. Appl. Poult. Res..

[7] Bailey RA, Watson KA, Bilgili SF, Avendano S (2015). The genetic basis of pectoralis major myopathies in modern broiler chicken lines. Poult. Sci..

[8] Alnahhas N (2016). Genetic parameters of White Striping in relation to body weight, carcass composition, and meat quality traits in two broiler lines divergently selected for the ultimate pH of the pectoralis major muscle. BMC Genet..

[9] Tixier-Boichard M (2020). From the jungle fowl to highly performing chickens: Are we reaching limits?. Worlds. Poult. Sci. J..

[10] Mutryn MF, Brannick EM, Fu W, Lee WR, Abasht B (2015). Characterization of a novel chicken muscle disorder through differential gene expression and pathway analysis using RNA-sequencing. BMC Genomics.

[11] Pampouille E (2018). Mapping QTL for White Striping in relation to breast muscle yield and meat quality traits in broiler chickens. BMC Genomics.

[12] Kang SW (2020). Characterization of stress response involved in chicken myopathy. Gen. Comp. Endocrinol..

[13] Malila Y (2020). Transcriptional profiles of skeletal muscle associated with increasing severity of White Striping in commercial broilers. Front. Physiol..

[14] Hoving-Bolink AH, Kranen RW, Klont RE, Gerritsen CLM, De Greef KH (2000). Fibre area and capillary supply in broiler breast muscle in relation to productivity and ascites. Meat Sci..

[15] Papah MB, Brannick EM, Schmidt CJ, Abasht B (2018). Gene expression profiling of the early pathogenesis of Wooden Breast disease in commercial broiler chickens using RNA-sequencing. PLoS ONE.

[16] Pampouille E (2019). Differential expression and co-expression gene network analyses reveal molecular mechanisms and candidate biomarkers involved in breast muscle myopathies in chicken. Sci. Rep..

[17] Sihvo HK, Immonen K, Puolanne E (2014). Myodegeneration with fibrosis and regeneration in the pectoralis major muscle of broilers. Vet. Pathol..

[18] Mazzoni M (2014). Relationship between pectoralis major muscle histology and quality traits of chicken meat. Poult. Sci.

[19] Velleman SG (2020). Pectoralis major (breast) muscle extracellular matrix fibrillar collagen modifications associated with the Wooden Breast fibrotic myopathy in broilers. Front. Physiol..

[20] Lake JA, Abasht B (2020). Glucolipotoxicity: A proposed etiology for Wooden Breast and related myopathies in commercial broiler chickens. Front. Physiol..

[21] Firtina Z (2009). Abnormal expression of collagen IV in lens activates unfolded protein response resulting in cataract. J. Biol. Chem..

[22] Lake JA, Papah MB, Abasht B (2019). Increased expression of lipid metabolism genes in early stages of wooden breast links myopathy of broilers to metabolic syndrome in humans. Genes.

[23] Cisternas P, Henriquez JP, Brandan E, Inestrosa NC (2014). Wnt signaling in skeletal muscle dynamics: Myogenesis, neuromuscular synapse and fibrosis. Mol. Neurobiol..

[24] Phillips CA, Reading BJ, Livingston M, Livingston K, Ashwell CM (2020). Evaluation via supervised machine learning of the broiler Pectoralis major and liver transcriptome in association with the muscle myopathy Wooden Breast. Front. Physiol..

[25] Ahmad K, Shaikh S, Ahmad SS, Lee EJ, Choi I (2020). Cross-talk between extracellular matrix and skeletal muscle: Implications for myopathies. Front. Pharmacol..

[26] Tam CS (2014). Weight gain reveals dramatic increases in skeletal muscle extracellular matrix remodeling. J. Clin. Endocrinol. Metab..

[27] Shan N (2015). Laminin α4 (LAMA4) expression promotes trophoblast cell invasion, migration, and angiogenesis, and is lowered in preeclamptic placentas. Placenta.

[28] Gatseva A, Sin YY, Brezzo G, Van Agtmael T (2019). Basement membrane collagens and disease mechanisms. Essays Biochem..

[29] Mohassel P, Foley AR, Bönnemann CG (2018). Extracellular matrix-driven congenital muscular dystrophies. Matrix Biol..

[30] Nguyen Q, Lim KRQ, Yokota T (2019). Current understanding and treatment of cardiac and skeletal muscle pathology in laminin-α2 chain-deficient congenital muscular dystrophy. Appl. Clin. Genet..

[31] Gawlik KI, Körner Z, Oliveira BM, Durbeej M (2019). Early skeletal muscle pathology and disease progress in the dy 3K/dy 3K mouse model of congenital muscular dystrophy with laminin α2 chain-deficiency. Sci. Rep..

[32] Davis MR, Summers KM (2012). Structure and function of the mammalian fibrillin gene family: Implications for human connective tissue diseases. Mol. Genet. Metab..

[33] Yalovaç A, Ulusu NN (2007). Collagen and collagen disorders. Fabad J. Pharm. Sci..

[34] Shin SJ, Yanagisawa H (2019). Recent updates on the molecular network of elastic fiber formation. Essays Biochem..

[35] Kuo DS, Labelle-Dumais C, Gould DB (2012). Col4a1 and col4a2 mutations and disease: Insights into pathogenic mechanisms and potential therapeutic targets. Hum. Mol. Genet..

[36] Meuwissen MEC (2015). The expanding phenotype of COL4A1 and COL4A2 mutations: Clinical data on 13 newly identified families and a review of the literature. Genet. Med..

[37] Sand JMB, Genovese F, Gudmann NS, Karsdal MA, Karsdal MA (2019). Type IV collagen. Biochemistry of Collagens, Laminins Elastin.

[38] Labelle-Dumais C (2019). COL4A1 mutations cause neuromuscular disease with tissue-specific mechanistic heterogeneity. Am. J. Hum. Genet..

[39] Mazzoni M (2020). Fiber metabolism, procollagen and collagen type III immunoreactivity in broiler pectoralis major affected by muscle abnormalities. Animals.

[40] Guiraud S (2017). HANAC Col4a1 mutation in mice leads to skeletal muscle alterations due to a primary vascular defect. Am. J. Pathol..

[41] Kuttappan VA, Owens CM, Coon C, Hargis BM, Vazquez-A Non M (2017). Incidence of broiler breast myopathies at 2 different ages and its impact on selected raw meat quality parameters. Poult. Sci..

[42] Soglia F (2020). Distribution and expression of vimentin and desmin in broiler Pectoralis major affected by the growth-related muscular abnormalities. Front. Physiol..

[43] Görlach A, Klappa P, Kietzmann T (2006). The endoplasmic reticulum: Folding, calcium homeostasis, signaling, and redox control. Antioxidants Redox Signal..

[44] Zhang IX, Raghavan M, Satin LS (2020). The endoplasmic reticulum and calcium homeostasis in pancreatic beta cells. Endocrinology.

[45] Williams AS, Kang L, Wasserman DH (2015). The extracellular matrix and insulin resistance. Trends Endocrinol. Metab..

[46] Baldi G (2020). Exploring the factors contributing to the high ultimate pH of vroiler Pectoralis major muscles affected by Wooden Breast condition. Front. Physiol..

[47] Papah MB, Brannick EM, Schmidt CJ, Abasht B (2017). Evidence and role of phlebitis and lipid infiltration in the onset and pathogenesis of Wooden Breast disease in modern broiler chickens. Avian Pathol..

[48] Langfelder P, Horvath S (2008). WGCNA: An R package for weighted correlation network analysis. BMC Bioinform..

[49] Huang D, Sherman B, Lempicki R (2009). Systematic and integrative analysis of large gene lists using DAVID bioinformatics resources. Nat. Protoc..

[50] Kanehisa M, Goto S (2000). KEGG: Kyoto encyclopedia of genes and genomes. Nucleic Acid Res..

[51] Bindea G (2009). ClueGO: A cytoscape plug-in to decipher functionally grouped gene ontology and pathway annotation networks. Bioinformatics.

[52] Shannon P (1971). Cytoscape: A software environment for integrated models. Genome Res..

[53] Chin CH (2014). cytoHubba: Identifying hub objects and sub-networks from complex interactome. BMC Syst. Biol..

