# Supplementary material for: Weighted gene co-expression network analysis identifies molecular pathways and hub genes involved in broiler White Striping and Wooden Breast myopathies
Source: Sci Rep. 2021 Jan 19;11:1776. doi: 10.1038/s41598-021-81303-7 (PMC7815844; doi:10.1038/s41598-021-81303-7)
Supplement: Supplementary file 1 — Supplementary Information. [file 41598_2021_81303_MOESM1_ESM.pdf]

Weighted gene co-expression network analysis identifies molecular pathways and hub genes involved in broilers' *White Striping* and *Wooden Breast* myopathies

Bordini Martina, Zappaterra Martina, Soglia Francesca, Petracci Massimiliano, Davoli Roberta

Supplementary Figure S1. Clustering dendrogram of the genes to construct the gene network and identify modules. The figure shows the cluster dendrogram of 18,803 probes considered for this analysis. Each branch in the figure represents one gene, and every color below represents one co-expression module.

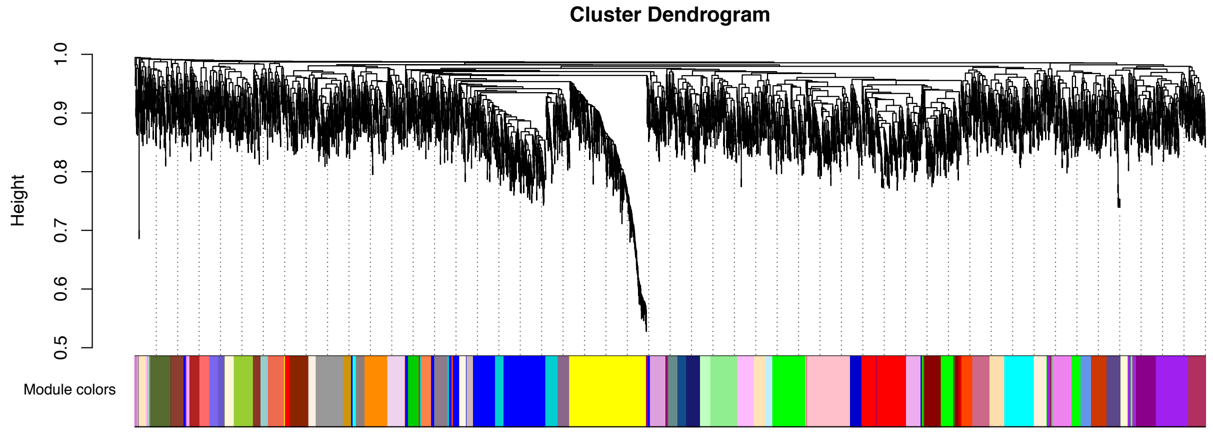

Supplementary Figure S2. Analysis of network topology for several soft-thresholding powers. The figure reports two graphs: the left graph shows the scale-free fit index as a function of the soft-thresholding power, and the right graph displays the mean connectivity as a function of the soft-thresholding power.

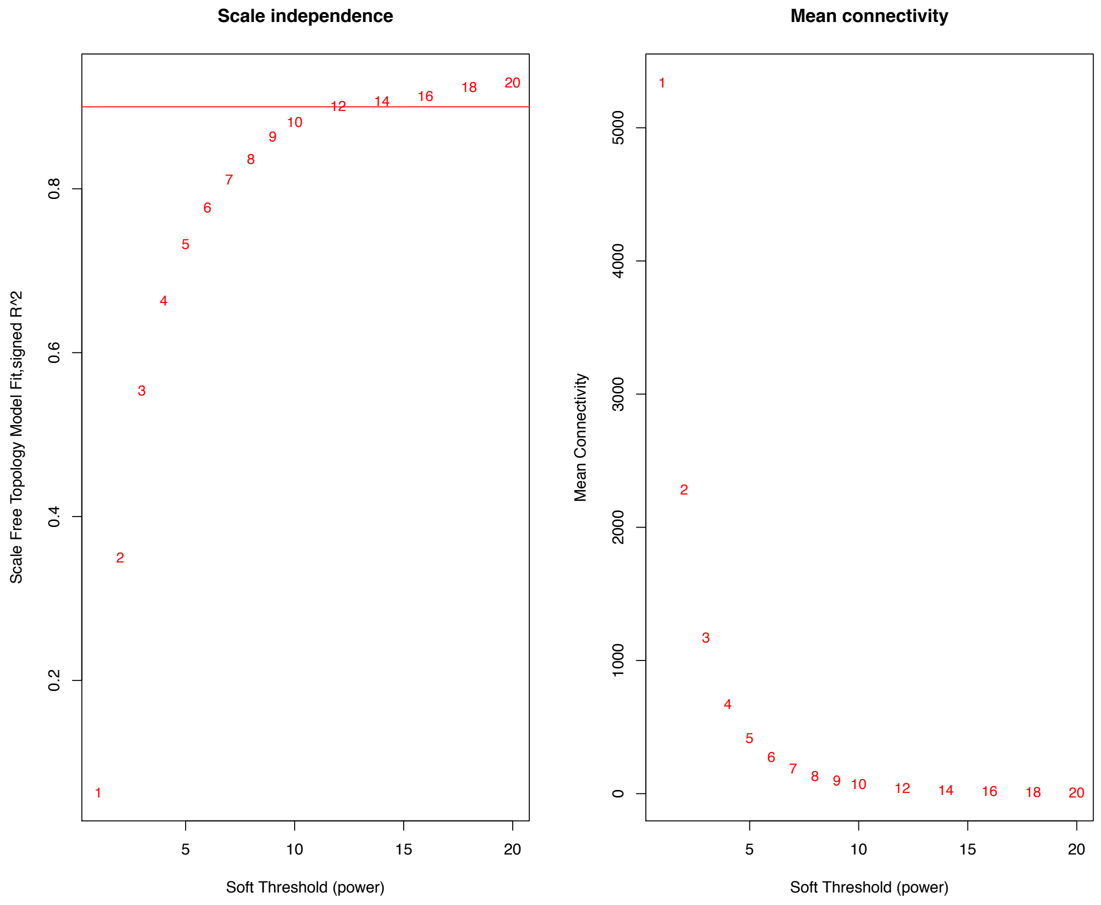

SUPPLEMENTARY TABLES CAPTIONS

Supplementary Table S1. Module-trait association values. Table reports Pearson's correlation coefficients measured between each single module and trait.

Supplementary Table S2. Macro-module and individual module Functional analysis performed using DAVID tools. The table reports the detailed results of the functional

classification of the macro-module and of each module analyses: darkred, midnightblue, paleturquoise4 and red.

Supplementary Table S3. Macro-module functional analysis performed using ClueGO Cytoscape plugin. The table reports macro-module functional categories identified by ClueGO.

Supplementary Table S4. Traits considered for our analysis to construct the network of co-expressed genes. Table reports all phenotypes analyzed by Zambonelli et al. [5] and that were considered for this study.

Supplementary Table S5. Measure of Module Significance in relation to the BW, H2.mm and CL traits. For each module, the MS values represent the average gene significance of all genes (absolute value) based on the correlation value between each module and the considered trait.

Supplementary Table S6. Gene significance (GS) of hub genes referred to traits belonging to the macro-trait, and the gene Module Membership values of the midnightblue, darkred, paleturquoise4 and red module. The supplementary table reports the GS values of genes identified as hub by CytoHubba plugin and the gene Module Membership values of modules selected for the analysis: midnightblue, darkred, paleturquoise4, and red.
